# Boerhaave Syndrome: An Unexpected Complication of Diabetic Ketoacidosis

**DOI:** 10.7759/cureus.25279

**Published:** 2022-05-24

**Authors:** Brandon Wiggins, Fady Banno, Kyle T Knight, Ian Fladie, Justin Miller

**Affiliations:** 1 Internal Medicine, Ascension Genesys Hospital, Grand Blanc, USA; 2 Internal Medicine, Baylor Scott & White Medical Center - Temple, Temple, USA; 3 Gastroenterology, Ascension Genesys Hospital, Grand Blanc, USA

**Keywords:** type 2 diabetes mellitus, esophagus rupture, diabetic ketoacidosis (dka), complications of dka, boerhaave’s syndrome

## Abstract

Boerhaave syndrome (BS) is a rare gastrointestinal condition related to esophageal rupture that carries a high mortality rate without prompt medical attention. BS is commonly associated with repeated episodes of severe retching, straining, or vomiting. Diabetic ketoacidosis (DKA), a serious acute complication of diabetes, is characterized in part by laboratory findings of profound hyperglycemia and ketoacidosis. Clinically, nausea and vomiting are seen commonly in DKA patients, which can often include repeated forceful retching, but rarely associated with esophageal rupture. In this article, we will describe a case of BS secondary to repeated episodes of emesis in the setting of DKA.

## Introduction

Boerhaave syndrome (BS) can be characterized by full-thickness esophageal perforation induced by barogenic trauma [1]. The mechanism for barogenic trauma involves repeat pyloric closure with subsequent diaphragmatic contraction against a closed cricopharyngeus [1]. Repeated increase of intra-esophageal pressure can then result in esophageal perforation [2,3]. Esophageal perforation in BS occurs in the left posterolateral aspect of the distal intrathoracic esophagus in 90% of patients. However, rupture of the cervical and intra-abdominal esophagus may also occur [1,4]. Rupture of the esophagus is subsequently followed by contamination of the mediastinum and pleural cavities by gastric contents; mechanical movement of the chest during respiration will dissipate these substances, leading to greater mediastinal and pleural soilage [1]. In the absence of medical and surgical intervention, bacterial infection and mediastinal necrosis will result, eventually leading to sepsis and multiple organ dysfunction syndrome [4,5].

BS in the setting of diabetic ketoacidosis (DKA) has been previously reported in the medical literature, albeit exceedingly rare. Upon review of available literature, two prior case reports describe such a situation. In one of these, a 2013 report by Alkuja et al., an iatrogenic etiology of esophageal rupture could not be ruled out [6,7].

## Case presentation

An otherwise healthy 22-year-old male presented to the emergency department due to nausea, vomiting, abdominal pain, and chest pain for the previous four days with associated fatigue and polyuria. Emesis was noted to be non-bilious, non-bloody but seemingly constant. The patient reported that he could not tolerate anything by mouth during this time. The chest pain was described as substernal, non-radiating, constant, progressively worsening, and provoked by eating. The abdominal pain was described as diffuse, non-radiating, constant, stabbing in nature, and provoked by eating.

Vital signs on arrival at the emergency department were significant for a heart rate of 108 beats per minute and a respiratory rate of 25 breaths per minute. Physical examination was remarkable for a lethargic male that appeared his stated age with tachypnea, tachycardia, and a diffusely tender abdomen in all four quadrants. Chest X-ray on presentation was read as a normal exam. Laboratory studies demonstrated glucose of 1347 mg/dL, anion gap of 50 mmol/L, and a white blood cell count of 26.3 k/cm^2^. Venous blood gas (VBG) revealed a pH of 7.149, pCO_2_ of 26 mmHg, and bicarbonate of 8.7 mmol/L, consistent with metabolic acidosis. Urinalysis (UA) showed 3+ glucose and 2+ ketones. Beta-hydroxybutyrate was over 8 mmol/L. Combined results of the VBG, UA, and comprehensive blood profile indicated that the patient was in DKA (Table 1). The patient was started on an intravenous (IV) insulin infusion and IV fluids and transferred to the intensive care unit. 

**Table 1:** Laboratory results on presentation

Laboratory Values	Measured	Normal Range
White Blood Cell Count	26.3 k/cm^2^	4.5-11 k/cm^2^
Hemoglobin	15.4 g/dL	11-16.2 g/dL
Hematocrit	50.6%	36-46%
Platelet Count	358 k/cm^2^	140-440 k/cm^2^
Serum Sodium Level	118 mmol/L	136-144 mmol/L
Serum Potassium Level	7 mmol/L	3.6-5.1 mmol/L
Serum Chloride Level	61 mmol/L	101-111 mmol/L
Total Serum Carbon Dioxide	7 mmol/L	20-30 mmol/L
Serum Blood Urea Nitrogen	59 mg/dL	8-26 mg/dL
Serum Creatinine Level	4.43 mg/dL	0.44-1.00 mg/dL
Anion Gap	50 mmol/L	8-16 mmol/L
Serum Glucose	1347 mg/dL	70-99 mg/dL
Serum Calcium Level	8.3 mg/dL	8.4-10.2 mg/dL
Serum Magnesium Level	3.6 mg/dL	1.6-2.6 mg/dL
Serum Phosphorus Level	14.9 mg/dL	2.3-4.7 mg/dL
Total Bilirubin	0.5 mg/dL	0.3-1 mg/dL
Serum Albumin Level	4.8 g/dL	3.5-5 g/dL
Aspartate Aminotransferase	8 U/L	15-41 U/L
Alanine Aminotransferase	18 U/L	14-54 U/L
Serum Alkaline Phosphatase	173 U/L	41-150 U/L
Beta Hydroxybutyrate	>8 mmol/L	0-0.3 mmol/L
Venous Blood Gas pH	7.149	7.32-7.42
Venous Blood Gas pCO_2_	26 mmHg	41-51 mmHg
Venous Blood Gas pO_2_	64 mmHg	37-43 mmHg
Venous Blood Gas Bicarbonate	8.7 mmol/L	23-29 mmol/L

**Figure 1:**
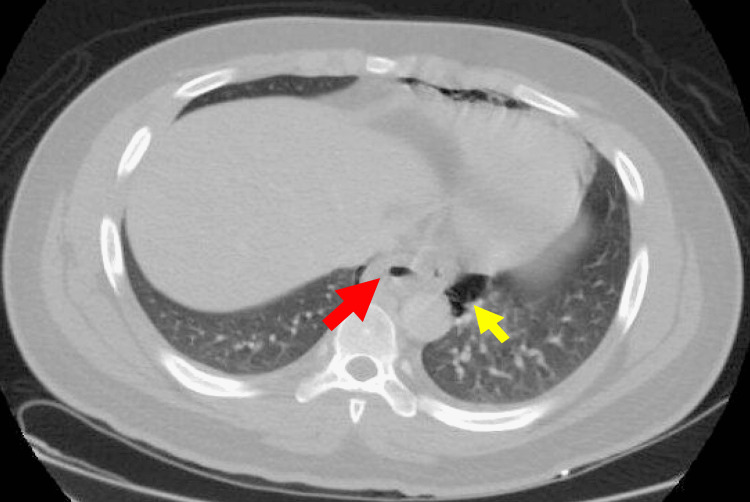
Transverse-view computed tomography of the chest in abdomen window Red arrow demonstrates thickening of the distal esophagus with perforation consistent with Boerhaave syndrome. Yellow arrow demonstrates pneumomediastinum secondary to esophageal perforation.

**Figure 2:**
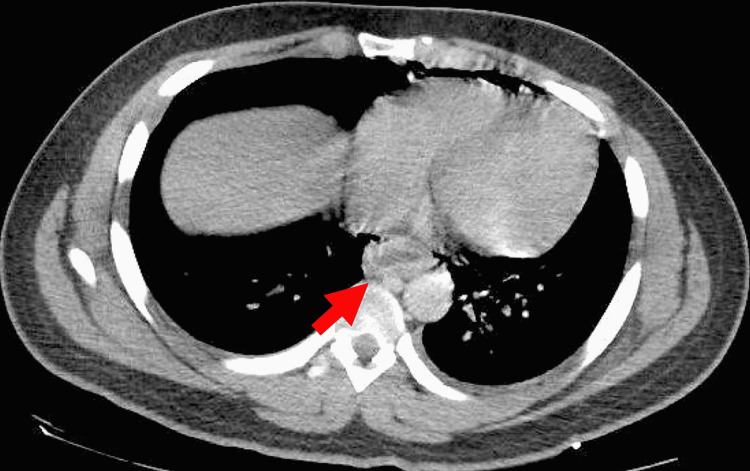
Transverse-view computed tomography of the chest in lung window Red arrow demonstrates esophageal thickening and perforation secondary to Boerhaave syndrome.

After transfer, the patient continued to complain of constant chest pain that was pleuritic. Computed tomography (CT) of the chest, abdomen, and pelvis with contrast was ordered due to concern for non-specific pain and demonstrated anterior and posterior pneumomediastinum with diffuse thickening of the distal esophagus, confirming esophageal perforation consistent with BS (Figures 1, 2). The patient was then transferred to a tertiary care facility with a dedicated cardiothoracic surgeon on call and lost to follow-up thereafter.

## Discussion

BS remains a feared complication secondary to sudden increased intra-esophageal pressure from retching with subsequent esophageal perforation. BS has an incidence rate of 3.1 per million and is fatal if left untreated [8]. BS typically is seen in middle-aged men between the ages of 50 and 70 and alcohol is usually involved [8].

BS usually occurs in patients without underlying esophageal disease. However, a subset of patients has esophageal rupture secondary to peptic ulcers, Barrett esophagus, eosinophilic esophagitis, pill esophagitis, and other causes of esophageal inflammation [2,3,8]. DKA has been associated with nausea, vomiting, and Mallory-Weiss tears in the extreme, but is seldomly associated with BS. BS typically is caused by a sudden increase in intra-esophageal pressure with an associated increase in intrathoracic pressure. Leakage of esophageal contents may cause chemical mediastinitis, predisposing patients to further complications including infection, mediastinal necrosis, pneumomediastinum, and end-organ damage [5].

Signs of BS vary widely based on the anatomical location of the rupture. Most commonly, patients present with refractory, unremitting chest pain, crepitus on palpation, fever, dyspnea, tachycardia, tachypnea, cyanosis, and hypotension [2]. More proximal ruptures can present with neck pain, dysphagia, or dysphonia [9].

BS is usually diagnosed incidentally in patients being evaluated for chest pain. It should be suspected in patients who complain of severe neck, chest, or upper abdominal pain after an episode of severe retching and vomiting. Other causes of increased intrathoracic pressure such as intubation, pneumothorax, and pleural effusions may also result in BS. Subcutaneous emphysema may be noted on physical examination and the diagnosis is established by CT scan with oral contrast. Delay in diagnosis is often associated with a higher risk of mortality and complications [10]. 

Myocardial infarction, pancreatitis, aortic aneurysm, peptic ulcer disease, pneumonia, or spontaneous pneumothorax can also cause acute-onset chest or upper abdominal pain. History, electrocardiogram, laboratory evaluation (e.g., cardiac biomarkers, D-dimer, pancreatic enzymes), diagnostic imaging (e.g. chest X-ray, abdominal ultrasound, CT chest/abdomen/pelvis), and physical examination are important to distinguish these etiologies from esophageal perforation. In addition, patients with Mallory-Weiss syndrome may have similar, albeit less severe presentation; however, there will be no evidence of subcutaneous, mediastinal, or peritoneal air on radiography or extravasation of esophageal contrast [11].

Perforations diagnosed within 12-24 hours have the best outcomes. There are three common treatment options including conservative, endoscopic, and surgical. Intensive care unit admission is recommended not only for patients with hemodynamic instability but also for patients with multiple comorbid conditions [12]. Additionally, all patients with esophageal perforation should avoid oral intake or nasogastric tube placement. IV broad-spectrum antibiotics, proton-pump inhibitors, and antiemetics should be initiated, and nutrition should typically be parenteral. Surgical consultation is warranted for all patients in case of further deterioration after medical or endoscopic management. 

Endoscopic management of esophageal perforation should be considered in patients who are not surgical candidates or with extensive underlying comorbidities [13-15]. A multidisciplinary team of endoscopists and thoracic surgeons may work together to deploy fully covered esophageal stents, through-the-scope clips, over-the-scope clips, endoscopic suturing, or esophageal resection and diversion.

The prognosis usually depends on the timing of diagnosis and treatment. Delayed diagnosis and treatment are usually associated with poor outcomes. Those who undergo diagnosis and surgery within 24 hours carry a survival rate of approximately 75%, but this can drop to 50% if the diagnosis and treatment are delayed after 24 hours and approximately 10% after 48 hours [16].

## Conclusions

BS related to DKA has rarely been described in medical literature in the past. With this rare association in mind, clinicians should add BS to the differential when faced with patients experiencing extreme chest pain in DKA. The severe retching seen in DKA typically manifests with Mallory-Weiss tears in the extremis, but BS has also been identified as a possible, albeit rare, complication discussed in the above case. This case was unique in the sense that many patients have severe retching during DKA but do not develop BS. Our patient also did not fit the typical demographic that develops BS other than being male, as he was not middle-aged or using alcohol.

In this case, we highlight the importance of radiologic imaging acquisition in a timely manner to provide a diagnosis for extreme chest pain in the setting of DKA. Also highlighted is the benefit of using CT chest and abdomen in DKA cases that present with chest pain and abdominal pain to rule in BS. Prompt CT will allow for appropriate surgical or endoscopic care quickly to prevent high morbidity and mortality associated with delay. We hope this case provides awareness to clinicians of BS as part of the differential diagnosis in patients with severe chest and abdominal pain after retching.
